# Rhizospheric Microbes and Nanoparticles Synergize to Enhance Plant Immune Responses

**DOI:** 10.1002/pei3.70183

**Published:** 2026-07-02

**Authors:** Mohammad Nazrul Islam Bhuiyan, Md. Saidur Rahman, Md. Mahfuzur Rahman, Barun Kanti Saha

**Affiliations:** ^1^ BCSIR Chattogram Laboratories, BCSIR Chattogram Bangladesh; ^2^ Institute of Food Science and Technology Bangladesh Council of Scientific and Industrial Research (BCSIR) Dhaka Bangladesh

**Keywords:** co‐delivery systems, nanobiotic synergies, nanoparticles, precision nanobiotics, rhizosphere microbes

## Abstract

Sustainable crop production increasingly requires innovative strategies that can enhance plant resilience while reducing dependence on synthetic agrochemicals. Recent advances in nanotechnology and rhizosphere microbiome research have created new opportunities to strengthen plant defense systems through integrated biological and material‐based approaches. This review introduces the concept of nanobiotic synergies, defined as the strategic combination of engineered or biologically synthesized nanoparticles with beneficial rhizospheric microorganisms to improve plant immunity, stress tolerance, nutrient acquisition, and overall crop performance. We critically examine the individual and interactive roles of nanoparticles and plant‐associated microbiomes in regulating immune signaling, rhizosphere communication, nutrient dynamics, and adaptation to biotic and abiotic stresses. Particular emphasis is placed on the mechanistic pathways underlying plant–microbe–nanoparticle interactions, including immune priming, modulation of root exudates, microbiome restructuring, antioxidant regulation, and stress‐responsive signaling networks. The review further evaluates emerging evidence supporting nanobiotic applications in disease suppression, stress mitigation, and sustainable crop management while addressing key challenges related to environmental safety, regulatory oversight, scalability, and long‐term ecosystem impacts. Finally, we propose a systems‐level framework integrating multi‐omics technologies, systems biology, and predictive computational approaches to guide the rational design of next‐generation nanobiotic agricultural inputs. Collectively, this review highlights the potential of nanobiotic strategies as a promising avenue for advancing climate‐resilient and environmentally sustainable agriculture.

## Graphical abstract

Nanobiotics integrate engineered nanoparticles with beneficial rhizosphere microbes to synergistically enhance plant immunity, nutrient acquisition, and stress resilience. Multi‐omics and AI‐driven approaches enable precision design, accelerating sustainable, climate‐smart agriculture, and resilient crop production.

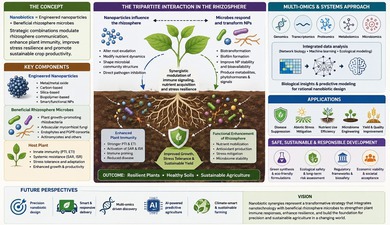

## 1 Introduction

Plants are continuously challenged by diverse biotic and abiotic stresses that adversely affect growth, productivity, and survival. To cope with these pressures, plants have evolved complex defense systems involving immune perception, phytohormone signaling, redox regulation, antimicrobial metabolite production, and systemic resistance mechanisms. However, the increasing prevalence of emerging pathogens, environmental degradation, climate change, and resource limitations continues to threaten agricultural sustainability and global food security (de Vries et al. 2020; Asghar et al. 2024; Muhammad et al. 2025).

Conventional crop protection strategies have relied heavily on synthetic fertilizers and pesticides, which have contributed significantly to agricultural productivity but have also generated concerns regarding soil degradation, biodiversity loss, environmental contamination, disruption of beneficial microbial communities, and the emergence of resistant pathogens (Rajput et al. 2021; Neme et al. 2021). These challenges have accelerated the search for sustainable approaches that enhance plant resilience while reducing ecological impacts.

Recent advances in nanotechnology and rhizosphere microbiome research have emerged as promising avenues for sustainable crop management. Nanoparticles possess unique physicochemical properties that enable targeted delivery, controlled release, enhanced reactivity, and stress‐mitigating functions, making them attractive candidates for nano‐enabled agricultural applications (Kumari et al. 2024; Ahmed et al. 2023). Simultaneously, beneficial rhizospheric microorganisms, particularly plant growth‐promoting rhizobacteria and arbuscular mycorrhizal fungi, contribute to nutrient acquisition, pathogen suppression, stress adaptation, and immune priming through diverse biochemical and ecological mechanisms (Montoya‐Martínez et al. 2022; Argentel‐Martínez et al. 2024; Behera et al. 2024).

Increasing evidence indicates that interactions between nanoparticles and rhizosphere microorganisms extend beyond their individual effects. Nanoparticles can influence microbial community composition, root exudation patterns, nutrient transformations, and signaling processes within the rhizosphere, whereas microorganisms can alter nanoparticle mobility, transformation, bioavailability, and biological activity (Guan et al. 2020; Ahmed et al. 2023; Prakash et al. 2024). These reciprocal interactions create opportunities for synergistic outcomes that may enhance plant defense, stress tolerance, and ecosystem functionality.

Within this context, the concept of nanobiotic synergies has emerged as a promising framework for integrating engineered or biologically synthesized nanoparticles with beneficial rhizospheric microorganisms to improve plant health and resilience. Unlike single‐component interventions, this approach seeks to combine the precision and multifunctionality of nanomaterials with the adaptive and self‐sustaining characteristics of beneficial microbiomes. Although recent studies have demonstrated encouraging outcomes in disease suppression, nutrient utilization, and stress mitigation, the mechanistic basis of nanoparticle–microbe–plant interactions, their long‐term ecological consequences, and their translational potential remain incompletely understood (Jiao et al. 2023; Gatasheh et al. 2024; Wei et al. 2025).

This review synthesizes current knowledge on the interactions among nanoparticles, rhizospheric microorganisms, and plant defense systems under the unifying framework of nanobiotic synergies. Particular emphasis is placed on the molecular, physiological, and ecological mechanisms governing nanoparticle–microbe–plant interactions, experimental evidence supporting their agricultural applications, environmental and biosafety considerations, and emerging opportunities arising from multi‐omics, systems biology, and predictive technologies. By identifying key knowledge gaps and future research priorities, this review aims to provide a conceptual foundation for the development of scientifically robust and environmentally sustainable nanobiotic strategies for next‐generation agriculture.

The specific objectives of this review are to: (i) examine the individual and combined contributions of nanoparticles and beneficial rhizospheric microorganisms to plant defense and resilience; (ii) elucidate the mechanistic pathways underlying nanoparticle–microbe–plant interactions; (iii) critically evaluate current evidence supporting nanobiotic applications in crop protection and stress management; and (iv) propose a future framework integrating multi‐omics, systems biology, biosafety assessment, and sustainable implementation strategies for precision agriculture.

## 2 Review Methodology

This review was conducted using a structured narrative‐review approach to synthesize current knowledge on the interactions among nanoparticles, rhizospheric microorganisms, and plant defense systems, with a particular focus on the emerging concept of nanobiotic synergies for sustainable agriculture. The methodology was designed to ensure transparency, comprehensiveness, and relevance while identifying major advances, knowledge gaps, and future research opportunities in this rapidly evolving field.

A systematic literature search was performed using major scientific databases, including Web of Science, Scopus, PubMed, and Google Scholar. Peer‐reviewed articles published primarily between 2020 and 2025 were prioritized to capture recent developments in nanotechnology, plant–microbe interactions, rhizosphere ecology, plant immunity, microbiome engineering, and sustainable agricultural systems. Additional seminal studies published before this period were included when considered essential for establishing foundational concepts. Search terms included combinations of keywords such as nanoparticles, nanotechnology in agriculture, rhizosphere microbiome, plant growth‐promoting rhizobacteria, arbuscular mycorrhizal fungi, plant immunity, induced systemic resistance, systemic acquired resistance, nano‐enabled agriculture, plant–microbe interactions, and sustainable crop protection.

Retrieved records were screened for relevance based on their direct contribution to understanding nanoparticle–microbe–plant interactions, defense‐related mechanisms, stress tolerance, nutrient dynamics, biosafety considerations, and agricultural applications. Studies lacking sufficient methodological detail, limited scientific rigor, or clear relevance to the review objectives were excluded. Priority was given to original research articles, high‐quality reviews, and recent advances providing mechanistic, ecological, or translational insights.

The selected literature was subsequently organized into thematic categories encompassing plant defense systems, rhizosphere microbiomes, nanoparticle‐mediated mechanisms, nanobiotic interactions, environmental and biosafety considerations, multi‐omics approaches, and future technological perspectives. A qualitative synthesis was then performed to identify recurring patterns, mechanistic relationships, emerging trends, current limitations, and future directions relevant to the development of next‐generation nanobiotic strategies for sustainable agriculture.

## 3 Plant Defense Systems: Foundations for Nanobiotic Synergies

Plants rely on sophisticated and interconnected defense networks to perceive, respond to, and adapt to biotic and abiotic stresses. These networks integrate immune signaling, phytohormone regulation, redox homeostasis, metabolic reprogramming, and plant–microbe interactions to maintain growth and survival under adverse conditions. Rather than functioning as independent pathways, plant defense responses emerge from coordinated communication among host tissues, associated microbiomes, and environmental cues, enabling plants to discriminate between beneficial and harmful organisms while balancing defense and development (Malgioglio et al. 2022; Saeed et al. 2021; Thepbandit and Athinuwat 2024).

The first layer of plant immunity is mediated by Pattern‐Triggered Immunity (PTI), which is activated when pattern‐recognition receptors detect conserved microbial signatures. PTI induces rapid defense responses, including calcium signaling, reactive oxygen species production, mitogen‐activated protein kinase activation, cell wall reinforcement, and defense‐gene expression. To overcome these barriers, pathogens secrete effector molecules that suppress host immunity. Plants, in turn, deploy intracellular nucleotide‐binding leucine‐rich repeat receptors that recognize pathogen effectors and activate Effector‐Triggered Immunity (ETI), a stronger defense response frequently associated with extensive transcriptional reprogramming and localized cell death (Saeed et al. 2021; Wahab et al. 2023).

Local immune responses are complemented by systemic defense mechanisms. Systemic Acquired Resistance is primarily associated with salicylic acid signaling and long‐term resistance following pathogen attack, whereas Induced Systemic Resistance is stimulated by beneficial rhizosphere microorganisms and is largely regulated through jasmonic acid‐ and ethylene‐dependent pathways (Dlamini et al. 2022; Montoya‐Martínez et al. 2022). Together, PTI, ETI, Systemic Acquired Resistance, and Induced Systemic Resistance form a multilayered immune architecture that enables plants to respond effectively to diverse environmental challenges (Table 1).

**TABLE 1:** Comparative overview of plant immune layers (PTI, ETI, SAR, ISR), signaling components, biological functions, and relevance to nanobiotic enhancement.

Plant immune layer	Primary trigger	Major signaling components	Principal biological responses	Functional role in plant defense	Potential relevance to nanobiotic enhancement	References
Pattern‐Triggered Immunity (PTI)	Recognition of conserved microbial signatures by pattern‐recognition receptors	Calcium influx, reactive oxygen species signaling, mitogen‐activated protein kinase cascades, defense‐related transcription factors	Cell‐wall reinforcement, antimicrobial metabolite production, oxidative burst, defense gene activation	First line of immune surveillance restricting pathogen establishment and colonization	Nanoparticles may enhance redox signaling, defense‐gene activation, root exudation dynamics, and microbiome‐mediated immune stimulation; beneficial microbes can amplify immune priming and signaling responsiveness	Saeed et al. (2021); Dlamini et al. (2022); Thepbandit and Athinuwat (2024); Ahmed et al. (2023)
Effector‐Triggered Immunity (ETI)	Recognition of pathogen effectors by intracellular nucleotide‐binding leucine‐rich repeat receptors	Nucleotide‐binding leucine‐rich repeat proteins, defense‐associated transcriptional networks, reactive oxygen species signaling	Strong immune activation, localized programmed cell death, pathogen containment, extensive transcriptional reprogramming	Provides robust and often race‐specific resistance against adapted pathogens	Nanoparticle‐mediated modulation of redox homeostasis and microbial interactions may reinforce ETI‐associated defense responses and pathogen suppression; mechanistic evidence remains limited and requires further validation	Wahab et al. (2023); Wang et al. (2025); Ahmed et al. (2023)
Systemic Acquired Resistance (SAR)	Localized pathogen infection and systemic immune signaling	Salicylic acid, pathogenesis‐related proteins, long‐distance defense signals	Long‐lasting systemic resistance against subsequent pathogen attack	Broad‐spectrum protection following infection and immune memory‐like responses	Nanoparticles functioning as defense elicitors may stimulate salicylic acid‐associated signaling, whereas beneficial microbiomes may support sustained systemic protection through improved physiological resilience	Montoya‐Martínez et al. (2022); Malgioglio et al. (2022); Ahmed et al. (2023)
Induced Systemic Resistance (ISR)	Colonization by beneficial rhizosphere microorganisms	Jasmonic acid, ethylene, microbial metabolites, volatile organic compounds	Immune priming, accelerated defense activation, enhanced stress responsiveness	Strengthens resistance without direct pathogen infection and links plant immunity to rhizosphere functions	Central target of nanobiotic strategies because nanoparticles can influence microbial recruitment, root exudation patterns, and rhizosphere communication, potentially enhancing microbe‐mediated immune priming	Dlamini et al. (2022); Montoya‐Martínez et al. (2022); Checcucci and Marchetti (2020); Thepbandit and Athinuwat (2024); Jiao et al. (2023); Jiao et al. (2025)
Holobiont‐level defense integration	Dynamic interactions among plant roots, microbiomes, environmental signals, and nanomaterials	Root exudates, microbial metabolites, phytohormones, nutrient signaling networks, redox regulators	Coordinated regulation of immunity, nutrient acquisition, stress adaptation, and microbiome assembly	Integrates local and systemic defense responses with rhizosphere ecological processes	Represents the conceptual foundation of nanobiotic agriculture, where nanoparticles and beneficial microbiomes jointly influence immune regulation, nutrient dynamics, and environmental resilience	de Vries et al. (2020); Ge and Wang (2025); Berruto and Demirer (2024); Ahmed et al. (2023); Zhang et al. (2024)

Abbreviations: ETI, Effector‐Triggered Immunity; ISR, Induced Systemic Resistance; PTI, Pattern‐Triggered Immunity; ROS, reactive oxygen species; SAR, Systemic Acquired Resistance.

The rhizosphere constitutes a critical extension of this defense system. Root‐associated microorganisms influence nutrient acquisition, hormone signaling, immune regulation, and stress adaptation through dynamic interactions with plant roots and root exudates. Beneficial microorganisms, particularly plant growth‐promoting rhizobacteria and arbuscular mycorrhizal fungi, enhance plant fitness through nitrogen cycling, phosphorus mobilization, pathogen suppression, antioxidant regulation, and immune priming (Amadou et al. 2021; Argentel‐Martínez et al. 2024; Behera et al. 2024). Recent studies demonstrate that arbuscular mycorrhizal fungi enhance drought resilience through coordinated regulation of nutrient acquisition, antioxidant defenses, and drought‐responsive molecular pathways, thereby improving plant physiological performance under environmental stress (Begum et al. 2023; Wang, Lian, et al. 2024; Wang, Zhang, et al. 2024).

Increasing evidence supports a holobiont perspective of plant defense in which immunity emerges from coordinated interactions between plants and their associated microbiomes. Microbial metabolites, volatile compounds, phytohormone analogs, and root exudate‐mediated communication collectively influence immune signaling, microbial community assembly, and stress adaptation (Checcucci and Marchetti 2020; Chen et al. 2022; Ge and Wang 2025). Consequently, the rhizosphere microbiome functions as an ecological extension of plant immunity, contributing to both pathogen resistance and environmental resilience.

Despite their complexity, plant defense systems are constrained by significant physiological and ecological trade‐offs. Defense activation requires substantial metabolic investment and may reduce growth and reproductive performance. Moreover, drought, flooding, salinity, heavy metal contamination, and pathogen evolution can disrupt immune regulation and rhizosphere functionality, reducing the effectiveness of natural defense mechanisms (de Vries et al. 2020; Francioli et al. 2021; Sun et al. 2024). These limitations highlight the need for complementary strategies capable of reinforcing endogenous defense networks while maintaining ecological sustainability.

### 3.1 Transition From Conventional Plant Immunity to Nanobiotic‐Enhanced Defense Systems

The integration of nanotechnology with beneficial rhizosphere microorganisms provides a promising framework for augmenting plant defense beyond conventional biological mechanisms. Rather than replacing natural immunity, nanobiotic strategies seek to strengthen existing plant–microbe interactions through the complementary functions of nanoparticles and beneficial microorganisms.

Emerging evidence indicates that nanoparticles influence plant defense by modulating redox homeostasis, nutrient availability, root exudation patterns, microbial community structure, and pathogen suppression (Guan et al. 2020; Ahmed et al. 2023; Zhang et al. 2024). Conversely, rhizosphere microorganisms can regulate nanoparticle transformation, mobility, bioavailability, and biological activity through biofilm formation, extracellular metabolites, and biogeochemical processes (Prakash et al. 2024). These reciprocal interactions create opportunities for synergistic enhancement of immune signaling, stress tolerance, and nutrient‐use efficiency.

Recent studies provide preliminary support for this concept. Selenium nanomaterials have been reported to influence root exudate–microbe interactions and stimulate plant growth through rhizosphere‐mediated processes, whereas cerium oxide nanomaterials modify communication between root exudates and rhizobacteria, contributing to improved crop performance (Jiao et al. 2023, 2025). Similarly, copper oxide nanoparticles can reshape rhizosphere microbial communities while influencing plant responses to biotic and abiotic stress (Guan et al. 2020; Wei et al. 2025). Although the underlying mechanisms remain incompletely resolved, these findings suggest that nanoparticles function not only as antimicrobial agents but also as regulators of plant–microbe communication networks.

**FIGURE 1:**
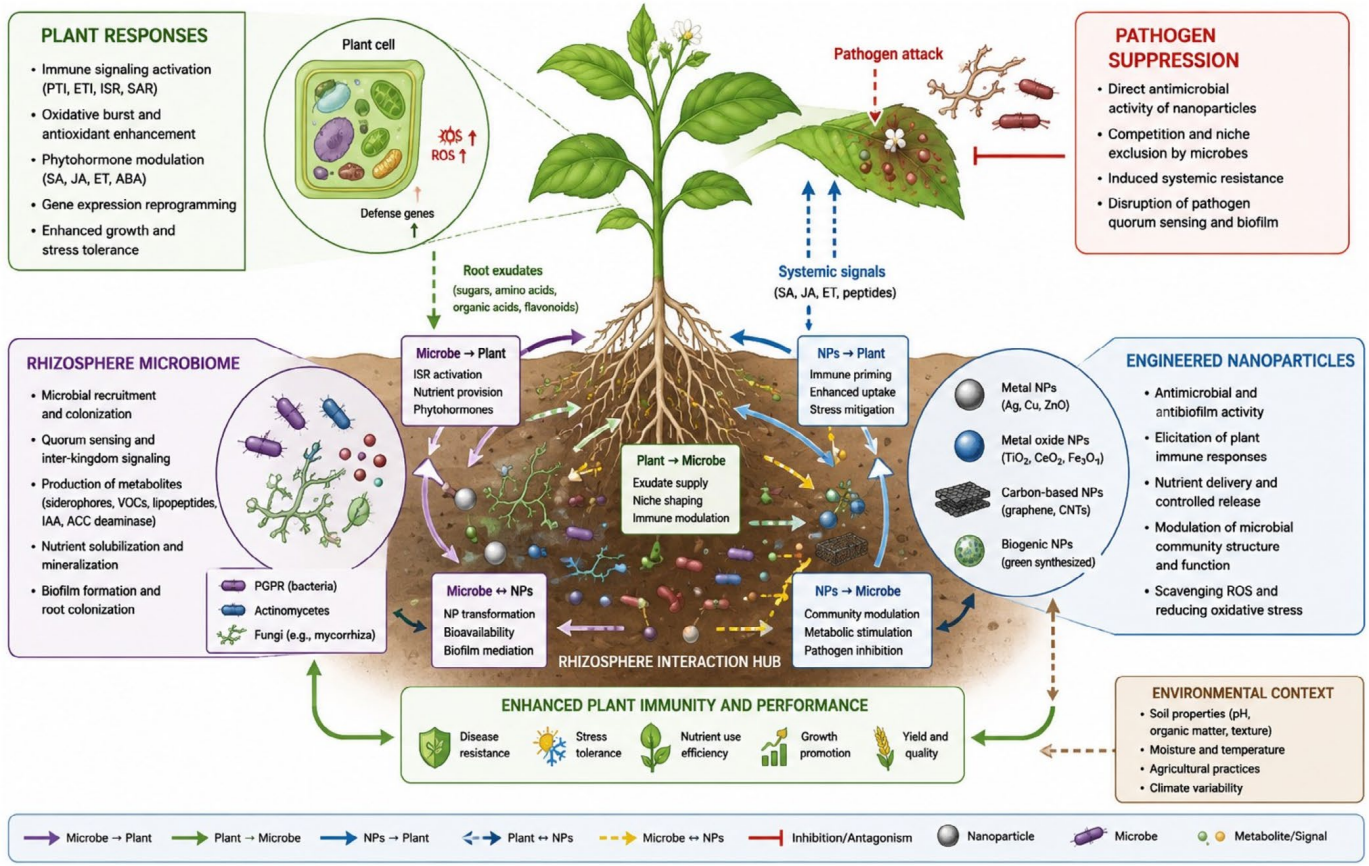
Multidirectional plant–microbe–nanoparticle interaction network underlying nanobiotic‐enhanced immunity.

Accordingly, Figure 1 presents a multidirectional interaction framework linking plants, root exudates, beneficial microorganisms, nanoparticles, and immune signaling pathways. This systems‐level perspective highlights the interconnected processes through which nanobiotic interventions may reinforce plant defense, improve stress resilience, and support sustainable crop production.

For a high‐impact review article, Sections 4 and 5 should be streamlined to avoid overlap, improve mechanistic focus, and strengthen the logical progression from nanoparticles → rhizosphere microbiome → nanobiotic synergies. The revision below reduces redundancy, emphasizes mechanisms rather than descriptions, and aligns with the editor's and reviewers' recommendations.

## 4 Nanoparticles as Modulators of Plant Immunity and Rhizosphere Function

Nanoparticles are increasingly recognized as multifunctional regulators of plant health, influencing immunity, stress adaptation, nutrient dynamics, and rhizosphere ecology. Their biological activity is governed by physicochemical characteristics such as composition, particle size, morphology, surface chemistry, concentration, and environmental transformation processes, which collectively determine their interactions with plants and soil microbiomes (Ahmed et al. 2023; Kumari et al. 2024; Zhang et al. 2024).

Among the most extensively studied nanomaterials in agriculture are silver, copper oxide, zinc oxide, iron oxide, selenium, and cerium oxide nanoparticles. These nanomaterials have demonstrated the capacity to suppress phytopathogens, enhance antioxidant defenses, improve nutrient acquisition, and influence rhizosphere microbial functions (Abd Alamer et al. 2021; Elsharkawy et al. 2020; Al‐Harethi et al. 2024; Liu et al. 2025). Furthermore, biologically synthesized nanoparticles produced using microorganisms or plant‐derived metabolites offer environmentally compatible alternatives to conventional synthesis approaches and support the development of sustainable nano‐enabled agricultural inputs (Abdelkhalek et al. 2022; Islam et al. 2024).

The effects of nanoparticles on plant defense extend beyond direct antimicrobial activity. Although membrane disruption, oxidative damage, and inhibition of pathogen metabolism contribute to pathogen suppression, increasing evidence indicates that nanoparticles also influence host defense through modulation of redox homeostasis, antioxidant systems, phytohormone signaling, and defense‐related gene expression (Kumari et al. 2024; Wei et al. 2025). Nanoparticle‐induced alterations in root exudation patterns further affect microbial recruitment and rhizosphere communication, indirectly shaping immune responses and stress adaptation (Ahmed et al. 2023; Jiao et al. 2025).

**FIGURE 2:**
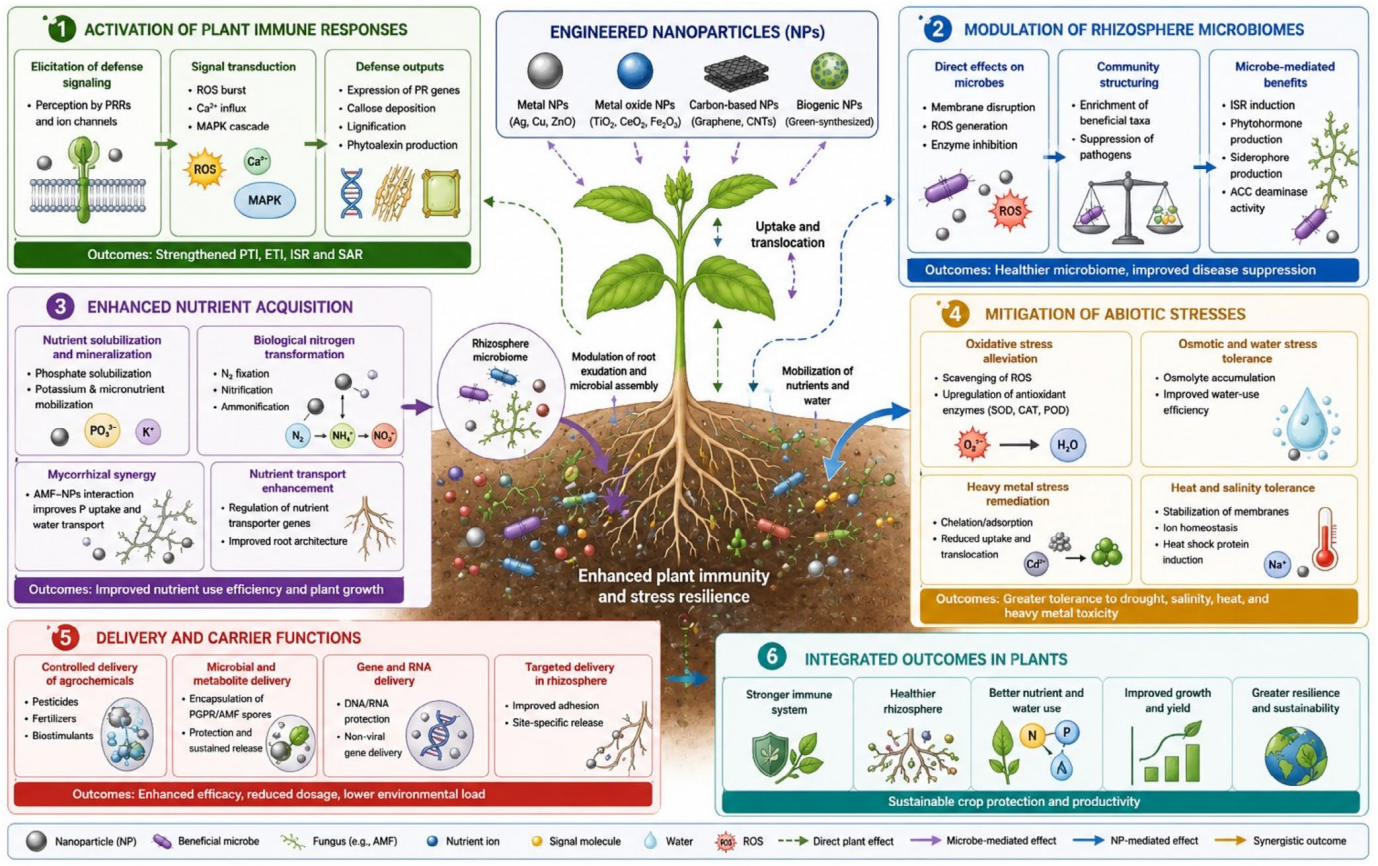
Mechanistic pathways through which nanoparticles modulate plant immunity, rhizosphere microbiomes, nutrient acquisition, and stress tolerance.

The rhizosphere is a major target of nanoparticle activity. Nanoparticles can influence microbial diversity, functional gene abundance, nutrient cycling, and ecological interactions within soil microbial communities (Guan et al. 2020; Zhang et al. 2024). Depending on nanoparticle properties and environmental conditions, these effects may either support beneficial microbiome functions or disrupt ecological balance. Consequently, nanoparticles should be viewed not merely as antimicrobial agents or delivery vehicles but as dynamic regulators of plant–microbe interactions that influence immunity, nutrient‐use efficiency, stress resilience, and rhizosphere functionality. The principal mechanisms through which nanoparticles regulate plant defense and rhizosphere processes are summarized in Figure 2.

## 5 Rhizosphere Microbiomes and Nanobiotic Synergies in Plant Defense

The rhizosphere is a highly dynamic biological interface where plant roots, microorganisms, and soil processes interact to regulate plant health and ecosystem functioning. Beneficial microorganisms contribute to nutrient acquisition, pathogen suppression, immune regulation, and environmental adaptation, making the rhizosphere microbiome a critical component of plant defense. Increasing evidence supports the concept that plant immunity extends beyond the host itself and emerges from coordinated interactions between plants and their associated microbial communities (de Vries et al. 2020; Dlamini et al. 2022; Ge and Wang 2025).

Plant growth‐promoting rhizobacteria and arbuscular mycorrhizal fungi represent the most extensively studied microbial groups involved in defense regulation. These organisms enhance plant performance through nitrogen fixation, phosphorus mobilization, siderophore production, phytohormone modulation, pathogen suppression, and induction of systemic resistance (Montoya‐Martínez et al. 2022; Argentel‐Martínez et al. 2024; Behera et al. 2024). Arbuscular mycorrhizal fungi further improve nutrient acquisition, water uptake, antioxidant metabolism, and stress‐responsive signaling, thereby enhancing plant tolerance to drought, heavy metal toxicity, and other environmental stresses (Begum et al. 2022, 2023; Chen, Sun, et al. 2025; Chen, Kama, et al. 2025).

A major contribution of beneficial microorganisms is their capacity to prime plant immunity. Through microbial metabolites, volatile compounds, and phytohormone‐mediated signaling, rhizosphere microorganisms enhance the responsiveness of defense pathways and facilitate more efficient activation of immune responses following pathogen attack or environmental stress (Dlamini et al. 2022; Thepbandit and Athinuwat 2024). In addition, microbial communities regulate root exudation patterns and influence microbiome assembly, contributing to the stability and functionality of beneficial rhizosphere networks (Checcucci and Marchetti 2020; Berruto and Demirer 2024).

### 5.1 Nanobiotic Synergies: Integrating Nanomaterials and Beneficial Microbiomes

The convergence of nanotechnology and rhizosphere microbiology has led to the emergence of nanobiotic synergies, a framework describing the coordinated use of nanoparticles and beneficial microorganisms to enhance plant defense, stress resilience, nutrient‐use efficiency, and agricultural sustainability. Unlike conventional approaches that employ these components independently, nanobiotic strategies seek to exploit their complementary and potentially synergistic functions within the plant–soil system (Ahmed et al. 2023; Prakash et al. 2024).

Nanoparticles can influence microbial recruitment, community composition, nutrient transformations, redox conditions, and root exudation patterns, thereby modifying the ecological environment in which beneficial microorganisms function (Guan et al. 2020; Ahmed et al. 2023). Emerging studies indicate that selenium and cerium oxide nanomaterials can modulate root exudate–microbiome interactions, leading to improved plant growth, rhizosphere functionality, and crop performance (Jiao et al. 2023, 2025). These findings suggest that nanoparticles act not only as antimicrobial agents but also as regulators of plant–microbe communication networks.

Reciprocally, microorganisms influence nanoparticle behavior through biofilm formation, extracellular polymeric substances, enzymatic transformations, and redox‐mediated processes that affect nanoparticle mobility, bioavailability, persistence, and biological activity (Prakash et al. 2024). These interactions create opportunities for designing biologically responsive nanomaterials and nano‐enabled microbial formulations capable of improving microbial survival, colonization efficiency, and functional stability under field conditions.

Despite these promising developments, nanobiotic interactions are highly context dependent. Inappropriate nanoparticle exposure may reduce microbial diversity, alter nutrient cycling processes, and disrupt beneficial ecological interactions (Guan et al. 2020; Ahmed et al. 2023; Zhang et al. 2024). Therefore, future nanobiotic technologies must prioritize ecological compatibility, optimized dosage regimes, green synthesis approaches, and long‐term biosafety evaluation.

Figure 2 illustrates the mechanistic pathways linking nanoparticles, root exudates, microbial communities, nutrient dynamics, immune signaling, and stress adaptation within the plant–microbe–nanoparticle continuum. This systems‐level framework highlights the multidirectional interactions that underpin nanobiotic synergies and provides the mechanistic basis for developing next‐generation sustainable agricultural technologies.

## 6 Experimental Evidence and Emerging Applications of Nanobiotic Strategies

Experimental evidence supporting nanobiotic strategies is expanding rapidly, highlighting the capacity of nanoparticle–microbe interactions to enhance plant immunity, stress resilience, rhizosphere functionality, and ecosystem sustainability. Although most studies remain confined to laboratory, greenhouse, or pot‐scale experiments, collectively they provide important mechanistic insights into how nanomaterials and beneficial microorganisms can interact to improve plant performance under diverse environmental conditions.

In plant disease management, biologically synthesized nanoparticles have demonstrated considerable antimicrobial potential against economically important phytopathogens. Silver nanoparticles produced by rhizosphere‐associated microorganisms inhibit bacterial and fungal pathogens while offering environmentally compatible alternatives to conventional chemical pesticides (Abd Alamer et al. 2021; Abdelkhalek et al. 2022). Similarly, biologically derived copper oxide nanoparticles suppress diseases caused by *Fusarium* spp. and *Phytophthora infestans* while simultaneously stimulating host defense responses (El‐Abeid et al. 2024; Al‐Harethi et al. 2024). As summarized in Table 2, these studies indicate that biologically synthesized nanomaterials can provide dual benefits by combining pathogen suppression with improved compatibility within beneficial rhizosphere environments.

**TABLE 2:** Experimental evidence for nanoparticle–microbe synergies in plant protection, stress mitigation, and rhizosphere engineering.

Nanoparticle system	Microbial partner/microbiome interaction	Crop or experimental system	Target stress or application	Proposed mechanistic basis	Major outcomes	Current limitations	References
Silver nanoparticles (biogenic)	Rhizosphere‐associated bacteria used for nanoparticle biosynthesis	Plant pathogen control systems	Bacterial wilt and other phytobacterial diseases	Antimicrobial activity combined with biologically mediated nanoparticle production; disruption of pathogen growth and colonization	Reduced pathogen proliferation and enhanced disease suppression potential	Predominantly laboratory‐scale evidence; limited field validation and microbiome impact assessment	Abd Alamer et al. (2021)
Silver nanoparticles (biogenic)	*Rhizobium leguminosarum* bv. *viciae*	Faba bean	Bean yellow mosaic virus infection	Rhizobial nanoparticle synthesis combined with antiviral and defense‐inducing activity	Reduced viral disease severity and improved plant performance	Long‐term ecological impacts and formulation stability remain unclear	Abdelkhalek et al. (2022)
Selenium nanomaterials	Rhizobacterial communities and root exudate interactions	Rice	Growth promotion and rhizosphere engineering	Modulation of root exudation patterns and stimulation of beneficial rhizosphere interactions	Enhanced plant growth and improved rhizosphere functionality	Mechanistic pathways require further molecular validation	Jiao et al. (2023)
Cerium oxide nanomaterials	Rhizobacteria–root exudate communication networks	Soybean	Yield and quality enhancement	Regulation of plant–microbe communication and rhizosphere metabolic processes	Increased crop productivity and quality traits	Evidence limited to specific crop systems	Jiao et al. (2025)
Copper oxide nanoparticles	Indigenous rhizosphere microbiome	Wheat rhizosphere	Rhizosphere engineering	Alteration of bacterial community composition and nitrogen‐cycling processes	Significant restructuring of rhizosphere microbial ecology	Ecological consequences remain context dependent	Guan et al. (2020)
Nano‐selenium	Indigenous rhizosphere microbiome	Potato	Potato scab disease and productivity improvement	Microbiome restructuring, pathogen suppression, and nutrient regulation	Enhanced disease resistance, yield, and tuber quality	Requires validation across soil types and environments	Liu et al. (2025)
Copper nanoparticles	Arbuscular mycorrhizal fungi	*Elymus sibiricus*	Arsenic toxicity	Metabolomic and ionomic reprogramming, reduced arsenic uptake, enhanced stress tolerance	Improved growth and arsenic stress mitigation	Limited field‐scale evaluation	Gatasheh et al. (2024)
Fullerenol nanoparticles	Arbuscular mycorrhizal fungi	*Brassica napus*	Lead toxicity	Antioxidant enhancement, physiological regulation, and stress‐response modulation	Improved tolerance to lead contamination	Long‐term nanoparticle persistence remains unknown	Shah, Usman, et al. (2024)
Silicon amendments with arbuscular mycorrhizal fungi	Arbuscular mycorrhizal fungi	*Brassica rapa*	Chromium toxicity	Regulation of chromium uptake, antioxidant defense, glyoxalase pathway, and secondary metabolism	Reduced toxicity and enhanced plant resilience	Requires agronomic validation under field conditions	Shah, Khan, Ali, et al. (2024)
Mycorrhizosphere‐associated bacteria	Beneficial rhizosphere bacterial consortium	Tomato	Chromium toxicity	Regulation of aquaporins, antioxidant enzymes, and proline metabolism	Reduced chromium accumulation and phytotoxicity	Mechanistic interactions with nanomaterials remain poorly understood	Shah, Khan, Alahmadi, et al. (2024)
Copper oxide nanoparticles	Rhizosphere microbiome	Soybean	*Fusarium* root rot disease	Activation of antioxidant defenses, regulation of isoflavone biosynthesis genes, and microbiome modulation	Enhanced disease resistance and improved plant performance	Long‐term microbiome stability requires investigation	Wei et al. (2025)
Green iron oxide nanoparticles	Indigenous microbial community	Loquat	*Fusarium* fruit rot disease	Direct pathogen suppression and stimulation of plant defense responses	Reduced disease incidence and improved fruit protection	Limited information on rhizosphere ecological effects	Niazi et al. (2023)

Abbreviation: AMF, arbuscular mycorrhizal fungi.

Among current applications, mitigation of abiotic stress represents one of the most promising areas of nanobiotic research. Integrated application of nanoparticles and arbuscular mycorrhizal fungi has enhanced plant performance under arsenic, chromium, and lead stress through coordinated regulation of antioxidant defenses, nutrient acquisition, ion homeostasis, osmotic adjustment, and metabolic reprogramming (Kumar 2021; Gatasheh et al. 2024; Shah, Usman, Noreen, et al. 2024; Shah, Khan, Ali, et al. 2024). Notably, the nanoparticle–arbuscular mycorrhizal fungus combinations presented in Table 2 consistently improved stress tolerance by modulating multiple physiological pathways simultaneously, supporting the central hypothesis that nanobiotic systems can generate multidimensional protective responses beyond those achieved by either component alone.

Emerging evidence further suggests that rhizosphere engineering is a key mechanism underlying nanobiotic functionality. Copper oxide nanoparticles have been shown to alter rhizosphere bacterial community composition and nitrogen‐cycling processes (Guan et al. 2020), whereas nano‐selenium applications influence microbiome structure, disease resistance, crop productivity, and crop quality (Liu et al. 2025). Similarly, selenium and cerium oxide nanomaterials modulate root exudate–microbiome interactions that promote nutrient utilization and plant growth in rice and soybean systems (Jiao et al. 2023, 2025). As highlighted in Table 2, these studies collectively demonstrate that nanoparticle‐induced changes in root exudation patterns, microbial recruitment, and microbiome functionality represent important mechanisms through which nanobiotic interventions enhance plant performance.

Beyond crop protection and productivity enhancement, nanobiotic approaches may also contribute to phytoremediation and ecosystem restoration. Although evidence from fully integrated nanoparticle–microbe systems remains limited, studies involving beneficial microbial symbioses indicate that coordinated interactions among plants and rhizosphere microorganisms can improve contaminant remediation, soil quality recovery, and vegetation establishment in degraded environments (Banerjee, Ghosh, et al. 2025; Banerjee, Jha, et al. 2025; Chen, Kama, et al. 2025). These findings broaden the potential applications of nanobiotic technologies beyond agricultural production and position them within wider sustainability, soil restoration, and environmental rehabilitation frameworks.

Despite encouraging progress, important limitations remain. Most studies employ different nanoparticle compositions, application methods, concentrations, microbial inoculants, crop species, and environmental conditions, making direct comparisons difficult and limiting mechanistic generalization. Furthermore, as summarized in Table 2, evidence remains heavily biased toward short‐term experimental systems, whereas long‐term ecological impacts, formulation stability, economic feasibility, and field‐scale performance remain insufficiently characterized. Consequently, although current findings strongly support the potential of nanobiotic approaches for plant protection and stress mitigation, large‐scale validation and mechanistic studies are still required before widespread agricultural implementation can be recommended.

Table 2 therefore provides a consolidated overview of experimentally validated nanoparticle–microbe combinations, target stresses, proposed mechanisms, crop responses, and current translational limitations. This evidence base highlights both the promise of nanobiotic technologies and the critical knowledge gaps that must be addressed to facilitate their transition from controlled experimental settings to sustainable field applications.

## 7 Environmental Sustainability and Biosafety of Nanobiotic Technologies

The future success of nanobiotic agriculture depends not only on biological efficacy but also on environmental compatibility, regulatory acceptance, and long‐term safety. Because nanomaterials interact simultaneously with plants, microorganisms, soil matrices, and biogeochemical processes, comprehensive evaluation of their ecological impacts is essential before large‐scale deployment (Ahmed et al. 2023; Rajput et al. 2021; Zhang et al. 2024).

### 7.1 Ecological Impacts on Plants and Rhizosphere Ecosystems

Nanoparticle effects are strongly influenced by physicochemical properties, dosage, exposure duration, and environmental context. While many studies report improved plant growth, stress tolerance, and disease resistance, excessive nanoparticle exposure may disrupt cellular homeostasis, impair root development, alter nutrient acquisition, and induce oxidative stress (Kumari et al. 2024; Zhang et al. 2024). Consequently, beneficial and detrimental outcomes frequently occur along a concentration‐dependent continuum.

The rhizosphere microbiome represents a particularly important target of nanoparticle exposure. Beneficial microbial communities regulate nutrient cycling, organic matter turnover, pathogen suppression, and immune priming. Alterations in microbial diversity or function may therefore influence ecosystem processes extending beyond individual crops (de Vries et al. 2020; Ahmed et al. 2023). For example, copper oxide nanoparticles have been shown to modify bacterial community composition and nitrogen‐cycling dynamics in agricultural soils (Guan et al. 2020). However, available evidence indicates that nanoparticle impacts are highly context dependent and cannot be generalized across nanoparticle classes or agroecosystems (Prakash et al. 2024; Zhang et al. 2024).

### 7.2 Environmental Fate, Risk Assessment, and Regulatory Challenges

A major knowledge gap concerns the environmental fate of nanoparticles after agricultural application. Nanoparticles may undergo aggregation, dissolution, adsorption, redox transformation, interaction with soil organic matter, or microbial modification, all of which influence mobility, persistence, and biological activity (Ahmed et al. 2023; Prakash et al. 2024). These transformation processes complicate environmental risk assessment and challenge conventional toxicological frameworks developed for bulk agrochemicals.

Current regulatory systems remain inadequately equipped to address nanoscale‐specific properties such as surface reactivity, transformation dynamics, and interactions with biological systems (Rajput et al. 2021; Rahman et al. 2025). Harmonized international standards for environmental monitoring, exposure assessment, lifecycle analysis, and biosafety evaluation are still lacking. Consequently, future risk‐assessment frameworks should adopt ecosystem‐level approaches integrating plant physiology, microbial ecology, soil processes, and environmental transformation pathways.

### 7.3 Green Synthesis and Sustainable Development Pathways

Environmentally compatible nanoparticle production represents a critical prerequisite for sustainable nanobiotic agriculture. Biogenic nanoparticles synthesized using bacteria, fungi, or plant‐derived biomolecules have attracted considerable interest because they reduce dependence on hazardous chemicals and may exhibit improved biocompatibility (Abd Alamer et al. 2021; Abdelkhalek et al. 2022; Islam et al. 2024). Such approaches also create opportunities to integrate microbial biotechnology and nanotechnology within circular bioeconomy frameworks.

Advances in nano‐encapsulation, controlled‐release formulations, and targeted delivery systems may further improve efficacy while minimizing environmental exposure and off‐target effects (Kumari et al. 2024; Rahman et al. 2025). Nevertheless, comprehensive lifecycle assessments and long‐term field studies remain necessary to determine whether these technologies provide net environmental benefits under realistic agricultural conditions.

**FIGURE 3:**
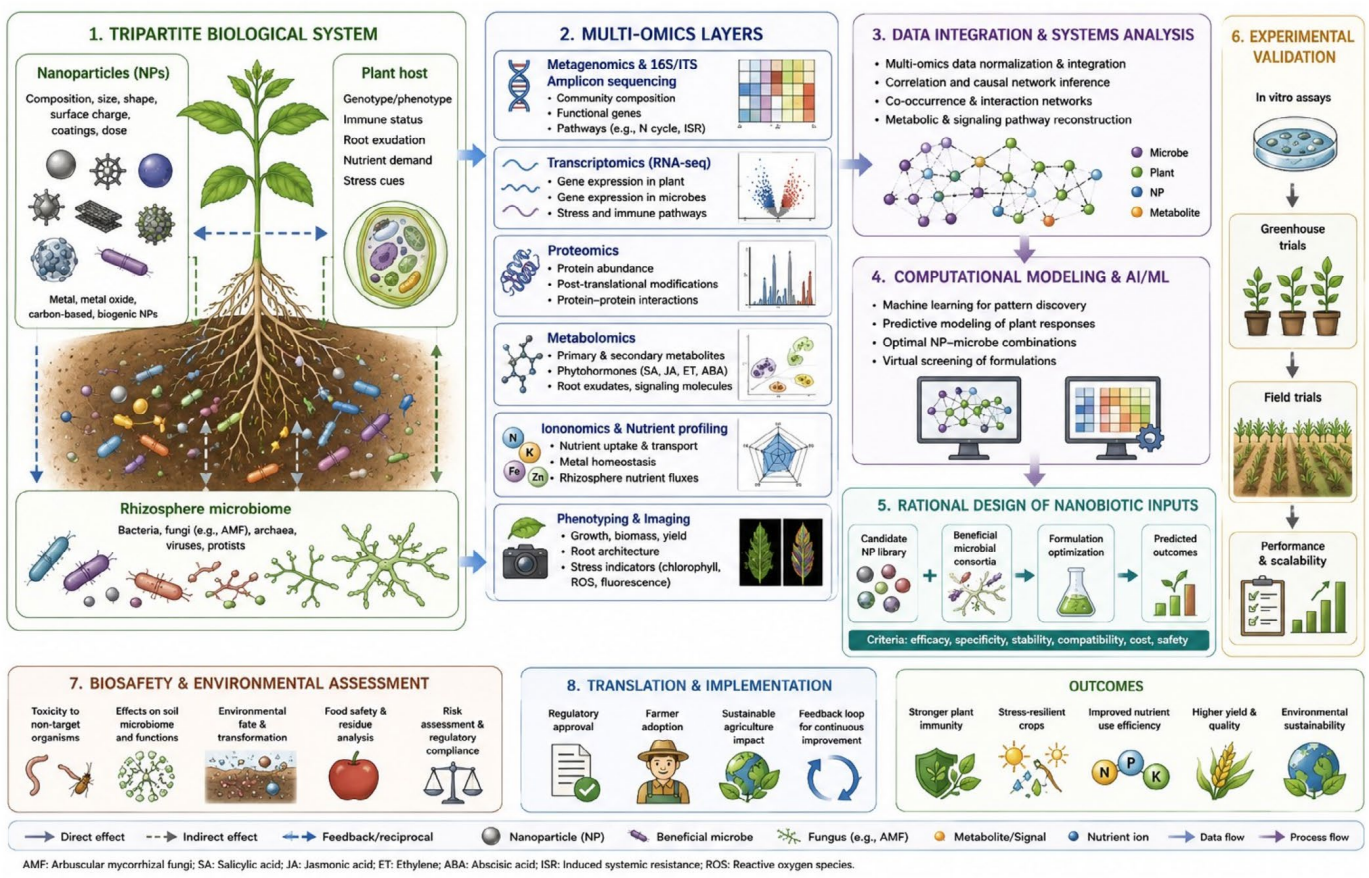
Multi‐omics and systems‐biology framework for rational design of next‐generation nanobiotic agricultural inputs.

Overall, environmental sustainability should be regarded as a central design criterion for future nanobiotic technologies. Consequently, Figure 3 integrates biosafety assessment, environmental monitoring, green synthesis, lifecycle evaluation, regulatory oversight, and systems‐level optimization within a unified framework for the development of next‐generation nanobiotic agricultural inputs.

For a high‐impact review article, Sections 8 and 9 should be integrated more tightly, avoid speculative overstatements, and explicitly position the proposed framework as a testable research model rather than a validated paradigm. The revision below addresses the editor's and reviewers' concerns regarding mechanistic depth, multi‐omics integration, systems biology, translational feasibility, and reduction of redundancy while maintaining a concise, Nature Reviews/Trends‐style narrative.

## 8 Current Research Gaps and Future Challenges

Despite growing interest in integrating nanotechnology and rhizosphere microbiome engineering, the scientific foundation of nanobiotic synergies remains at an early stage of development. Existing studies demonstrate considerable potential for enhancing plant immunity, stress tolerance, nutrient‐use efficiency, and ecosystem resilience; however, several fundamental barriers continue to limit translation from experimental observations to practical agricultural applications.

### 8.1 Mechanistic Knowledge Gaps Across the Plant–Microbe–Nanoparticle Continuum

Despite growing evidence supporting the benefits of nanobiotic interventions, the mechanistic basis of interactions within the plant–microbe–nanoparticle continuum remains insufficiently understood. Most studies primarily report phenotypic outcomes, such as enhanced plant growth, improved stress tolerance, disease suppression, or altered microbial community composition, whereas the underlying molecular, biochemical, and ecological processes responsible for these responses remain poorly resolved (Ahmed et al. 2023; Berruto and Demirer 2024). Consequently, critical questions persist regarding how nanoparticles influence root exudation dynamics, microbial communication networks, microbiome assembly, nutrient exchange processes, immune priming, and stress‐responsive signaling pathways within the rhizosphere.

A further challenge arises from the inherently multiscale nature of nanobiotic interactions. Plant responses are governed not only by direct nanoparticle effects but also by complex feedback mechanisms involving microbial communities, soil physicochemical properties, and environmental conditions. These interactions extend from molecular and cellular processes to community‐level and ecosystem‐scale responses, making mechanistic interpretation particularly challenging. As a result, current knowledge remains fragmented, limiting the development of predictive frameworks for nanobiotic system design and optimization.

Addressing these knowledge gaps will require integrated, systems‐level approaches that combine genomics, transcriptomics, proteomics, metabolomics, and microbiome profiling with advanced computational analyses. In particular, multilayer network analysis, machine learning, ecological modeling, metabolic flux analysis, and systems‐biology frameworks offer powerful tools for identifying causal relationships, regulatory hubs, and emergent properties within complex plant–microbe–nanoparticle networks. Such approaches can move the field beyond descriptive observations toward a mechanistic understanding of how nanobiotic interactions influence plant immunity, nutrient acquisition, stress adaptation, and rhizosphere functionality.

As summarized in Table 3, each omics platform provides complementary insights into nanobiotic systems, ranging from microbial community dynamics and gene‐expression responses to protein regulation and metabolite‐mediated communication. Table 3 also highlights important analytical challenges associated with nanoparticle‐containing biological samples, including nanoparticle–protein and nanoparticle–metabolite corona formation, which may influence extraction efficiency, analytical accuracy, and data interpretation. Collectively, these multi‐omics approaches provide the mechanistic foundation necessary for developing predictive models and enabling the rational design of next‐generation nanobiotic technologies for sustainable agriculture.

**TABLE 3:** Multi‐omics approaches (genomics, transcriptomics, proteomics, metabolomics, microbiomics), measured variables, biological insights, analytical challenges, and applications in nanobiotic system design.

Omics layer	Primary biological targets	Representative measured variables	Biological insights for nanobiotic systems	Major analytical challenges in nanoparticle‐containing systems	Potential applications in rational nanobiotic design	References
Genomics	Plant, microbial, and rhizosphere genetic potential	Functional genes, stress‐resistance genes, nutrient‐cycling genes, microbial community gene inventories	Identifies genetic determinants of plant resilience, microbiome functionality, nutrient mobilization, and stress adaptation	Distinguishing active versus dormant genetic potential; limited functional validation of candidate genes	Selection of microbial inoculants possessing desirable nutrient acquisition, pathogen suppression, or stress‐resistance traits	Berruto and Demirer (2024); Ge and Wang (2025); Wang et al. (2025)
Transcriptomics	Dynamic gene‐expression responses in plants and microorganisms	Differentially expressed genes, defense‐related transcripts, signaling pathways, transporter genes, stress‐responsive regulators	Reveals molecular responses to nanoparticle exposure, microbiome recruitment, immune activation, and environmental stress	Temporal variability; RNA degradation; difficulty distinguishing direct nanoparticle effects from secondary biological responses	Identification of regulatory pathways targeted by nanoparticle–microbe interactions and optimization of formulation timing	Begum et al. (2023); Huang et al. (2020); Han et al. (2022); Wang, Zhang, et al. (2024)
Proteomics	Functional protein networks and cellular machinery	Enzymes, transport proteins, signaling proteins, antioxidant proteins, defense‐related proteins	Characterizes functional responses beyond gene expression and identifies pathways directly involved in stress mitigation and immunity	Nanoparticle–protein corona formation may interfere with protein extraction, separation, and mass spectrometric analyses	Discovery of protein biomarkers associated with successful nanobiotic responses and stress resilience	Kumari et al. (2024); Ahmed et al. (2023)
Metabolomics	Plant, microbial, and rhizosphere metabolites	Phytohormones, secondary metabolites, root exudates, osmoprotectants, antioxidant compounds, signaling molecules	Provides direct evidence of biochemical interactions underlying immune priming, nutrient acquisition, and stress adaptation	Nanoparticle–metabolite interactions and adsorption may alter extraction efficiency and analytical accuracy	Identification of metabolic signatures guiding formulation design and optimization of nanoparticle–microbiome combinations	Gatasheh et al. (2024); Jiao et al. (2023); Jiao et al. (2025); Nie et al. (2024)
Microbiomics (16S rRNA/ITS sequencing, metagenomics)	Rhizosphere, endosphere, and soil microbial communities	Community composition, diversity indices, microbial networks, functional taxa, metagenomic profiles	Reveals how nanoparticles influence microbiome assembly, microbial recruitment, ecosystem stability, and pathogen suppression	Correlation does not necessarily imply function; strong environmental influences on microbial community composition	Identification of beneficial microbial consortia compatible with specific nanoparticle formulations	Ahmed et al. (2023); Guan et al. (2020); Chen, Sun, et al. (2025); Ge and Wang (2025)
Ionomics	Elemental composition and nutrient homeostasis	Mineral nutrients, trace elements, toxic metals, nutrient‐use efficiency indicators	Clarifies nanoparticle effects on nutrient uptake, metal detoxification, and physiological adaptation	Distinguishing nanoparticle‐derived elements from endogenous nutrient pools	Optimization of nano‐enabled nutrient delivery systems and remediation strategies	Gatasheh et al. (2024); Shah, Khan, Ali, et al. (2024); Kumar (2021)
Integrated multi‐omics	Cross‐scale biological networks linking plants, microbiomes, and nanomaterials	Combined genomic, transcriptomic, proteomic, metabolomic, microbiomic, and environmental datasets	Reveals causal relationships, emergent properties, signaling networks, and system‐level responses within nanobiotic interactions	Data integration complexity, standardization challenges, computational requirements, and heterogeneous data formats	Enables predictive design of crop‐specific nanobiotic formulations and precision agricultural interventions	Berruto and Demirer (2024); Ge and Wang (2025); Ahmed et al. (2023); Muhammad et al. (2025)

Abbreviations: ITS, internal transcribed spacer; rRNA, ribosomal ribonucleic acid.

### 8.2 Limited Field Validation and Translational Readiness

Another major challenge is the scarcity of long‐term field‐based investigations. Most available evidence originates from controlled laboratory, hydroponic, greenhouse, or pot experiments where environmental variability is minimized. While such studies provide valuable mechanistic insights, they do not adequately represent the complexity of agricultural ecosystems characterized by fluctuating climatic conditions, heterogeneous soils, diverse microbiomes, and multiple interacting stressors (Ahmed et al. 2023; Muhammad et al. 2025).

Consequently, the reproducibility, stability, and scalability of many reported nanobiotic effects remain uncertain. Future studies should prioritize multi‐location field trials that evaluate formulation performance, ecological compatibility, persistence, and economic feasibility under realistic production systems. Such validation is critical for determining whether experimental successes can be translated into commercially viable technologies.

### 8.3 Long‐Term Ecological and Food‐Safety Implications

The long‐term consequences of repeated nanoparticle application within agricultural systems remain poorly understood. Key uncertainties include nanoparticle accumulation in soil, interactions with soil organic matter, impacts on beneficial microbial communities, movement through food webs, and potential implications for crop quality and food safety (Guan et al. 2020; Zhang et al. 2024). At present, available evidence remains fragmented and insufficient to support definitive conclusions regarding long‐term ecological outcomes.

Addressing these uncertainties will require coordinated longitudinal studies integrating environmental monitoring, microbiome analyses, plant physiological assessments, and food‐safety evaluations. Such investigations are particularly important for detecting cumulative effects that may not be evident in short‐term experiments.

### 8.4 Economic, Regulatory, and Societal Barriers

Beyond scientific challenges, successful implementation of nanobiotic technologies depends on economic feasibility, regulatory acceptance, and societal trust. Production costs remain a significant limitation for many nano‐enabled agricultural products, highlighting the importance of scalable green synthesis approaches based on biological systems and renewable resources (Islam et al. 2024; Rahman et al. 2025).

Regulatory uncertainty further complicates technology development and commercialization. Existing frameworks were largely designed for conventional agrochemicals and frequently fail to account for nanoscale properties, environmental transformation processes, and microbiome‐mediated interactions. Equally important is public acceptance, which will depend on transparent risk communication, rigorous safety assessments, and evidence‐based regulatory oversight.

## 9 Toward a Systems‐Level Framework for Nanobiotic Agriculture

### 9.1 A Testable Conceptual Framework for Nanobiotic Synergies

Current evidence indicates that nanoparticles and beneficial rhizosphere microorganisms can independently improve plant immunity, nutrient acquisition, and stress adaptation. Nanoparticles influence nutrient dynamics, redox homeostasis, microbial community structure, and pathogen suppression, whereas beneficial microorganisms regulate immune priming, nutrient mobilization, microbiome stability, and stress‐responsive signaling pathways (Ahmed et al. 2023; Argentel‐Martínez et al. 2024; Behera et al. 2024). However, the extent to which their combined application consistently generates synergistic outcomes remains insufficiently established.

Based on available evidence, we propose the following testable hypothesis: “Strategically designed nanoparticle–microbiome combinations can enhance plant defense and stress resilience by simultaneously modulating rhizosphere communication, microbial functionality, nutrient transformations, and immune signaling pathways, resulting in integrated biological responses that exceed those achieved by individual interventions.”

Importantly, this framework should be regarded as a conceptual model for future investigation rather than a validated biological principle. Although several studies report beneficial interactions between nanoparticles and rhizosphere microorganisms, robust evidence demonstrating consistent synergistic effects across diverse crops, environments, and stress scenarios remains limited.

### 9.2 Mechanistic Pillars of the Nanobiotic Framework

The proposed framework conceptualizes the rhizosphere as a multidirectional communication network linking plants, microorganisms, nanomaterials, and environmental processes. Within this network, nanobiotic interactions are hypothesized to emerge through three interconnected mechanistic pillars.

#### 9.2.1 Rhizosphere Communication and Immune Priming

Root exudates regulate microbial recruitment, nutrient mobilization, and signaling exchanges within the rhizosphere. Nanoparticles can influence exudation patterns and microbiome assembly, thereby indirectly affecting plant defense pathways (Ahmed et al. 2023; Jiao et al. 2023, 2025). Concurrently, beneficial microorganisms produce signaling compounds, phytohormone analogs, and bioactive metabolites that contribute to immune priming and stress adaptation (Checcucci and Marchetti 2020; Dlamini et al. 2022). The integration of these processes may enhance plant responsiveness to subsequent biotic and abiotic challenges.

#### 9.2.2 Functional Optimization of Rhizosphere Processes

Nanoparticles may improve nutrient availability, delivery efficiency, and stress‐mitigating biochemical processes within the rhizosphere. Simultaneously, microorganisms influence nanoparticle mobility, transformation, aggregation, and bioavailability through biofilms, extracellular metabolites, and redox‐mediated processes (Prakash et al. 2024). These reciprocal interactions create opportunities for improving nutrient acquisition, antioxidant capacity, and physiological resilience, although the extent of true synergism requires further validation.

#### 9.2.3 Integrated Stress Resilience

A potential advantage of nanobiotic systems is the simultaneous modulation of multiple protective mechanisms. Rather than targeting individual pathways, integrated nanoparticle–microbiome interventions may influence immune signaling, oxidative stress regulation, nutrient uptake, microbial community stability, and adaptive physiological responses concurrently. Such multidimensional regulation may be particularly valuable under field conditions where crops are exposed to multiple interacting environmental stresses (Muhammad et al. 2025; Ge and Wang 2025).

Collectively, these interactions support a systems‐level perspective in which plant defense emerges from coordinated activity across the plant–microbe–nanoparticle continuum rather than from isolated biological components.

### 9.3 Future Directions: From Descriptive Studies to Predictive Nanobiotic Design

The next phase of nanobiotic research should transition from descriptive characterization toward predictive and mechanistically informed design. Advances in multi‐omics technologies, microbiome engineering, systems biology, and artificial intelligence provide unprecedented opportunities to identify biomarkers, predict functional interactions, and optimize nanoparticle–microbe combinations before field deployment (Berruto and Demirer 2024; Ge and Wang 2025).

A particularly promising direction involves integrating genomics, transcriptomics, proteomics, metabolomics, and microbiome datasets through machine‐learning and network‐based approaches to identify causal mechanisms and guide rational formulation design. Such strategies could accelerate the development of next‐generation biofertilizers, biostimulants, and bioprotectants while reducing empirical trial‐and‐error experimentation.

Accordingly, Figure 3 should serve as the central integrative framework of the review, illustrating interactions among plant immune signaling, root exudate‐mediated communication, microbiome assembly, nanoparticle transformation, multi‐omics data generation, predictive modeling, biosafety assessment, and precision agricultural deployment. Integrating these elements into a single systems‐biology framework improves conceptual coherence, addresses reviewer recommendations regarding multidirectional interactions, and eliminates the need for an additional conceptual figure.

## 10 Future Perspectives: Advancing Toward Precision Nanobiotic Agriculture

The convergence of nanotechnology, microbiome engineering, systems biology, and digital agriculture is creating unprecedented opportunities for the development of next‐generation crop protection and resilience strategies. However, translating nanobiotic synergies from an emerging concept into a deployable agricultural technology requires substantial advances in mechanistic understanding, formulation optimization, biosafety assessment, and field‐scale validation. Future research should therefore prioritize predictive, environmentally sustainable, and crop‐specific nanobiotic systems capable of functioning reliably across diverse agroecosystems. The complementary contributions of genomics, transcriptomics, proteomics, metabolomics, and microbiomics to elucidating plant–microbe–nanoparticle interactions are summarized in Table 3.

A key priority is the transition from generalized formulations toward precision nanobiotic interventions tailored to crop genotype, rhizosphere microbiome composition, soil characteristics, and prevailing environmental stressors. Because nanoparticle–microbe–plant interactions are highly context dependent, microbiome‐informed formulation strategies may enhance efficacy while minimizing unintended ecological impacts. Such approaches align closely with the principles of precision agriculture, in which biological inputs are optimized according to local environmental and agronomic conditions.

The development of smart and responsive delivery systems represents another major frontier. Advances in nano‐encapsulation, controlled‐release technologies, and stimuli‐responsive materials may enable targeted delivery of nutrients, microbial inoculants, signaling molecules, and bioprotective agents in response to environmental cues such as drought, nutrient limitation, pathogen attack, or changes in rhizosphere chemistry. These innovations have the potential to improve resource‐use efficiency, enhance formulation stability, and reduce off‐target environmental exposure.

Future breakthroughs will depend heavily on the integration of multi‐omics platforms with systems‐biology frameworks. Combining genomic, transcriptomic, proteomic, metabolomic, microbiomic, and environmental datasets can provide a comprehensive understanding of the molecular and ecological networks underlying nanobiotic functionality. In particular, multilayer network analysis, metabolic modeling, ecological simulation, and machine‐learning approaches offer powerful tools for identifying regulatory hubs, predictive biomarkers, and emergent interactions that are difficult to resolve through conventional experimentation alone. Future studies should also address methodological challenges associated with nanoparticle‐containing biological samples, including nanoparticle–protein and nanoparticle–metabolite corona formation, which can influence extraction efficiency, analytical sensitivity, and interpretation of omics data.

Artificial intelligence is expected to play an increasingly important role in accelerating nanobiotic innovation. Integration of multi‐omics datasets, microbiome profiles, environmental variables, and crop‐performance indicators could facilitate predictive modeling of plant–microbe–nanoparticle interactions and support rational formulation design. Although current applications remain largely exploratory, artificial intelligence‐assisted decision‐support systems may ultimately enable site‐specific optimization of nanobiotic interventions across diverse agricultural environments while reducing development costs and experimental uncertainty.

From a sustainability perspective, nanobiotic technologies offer considerable potential for advancing climate‐smart agriculture. By improving nutrient‐use efficiency, enhancing tolerance to drought, salinity, and emerging pathogens, supporting beneficial microbiomes, and reducing reliance on synthetic agrochemicals, these approaches may contribute to more resilient and environmentally sustainable production systems. Nevertheless, large‐scale implementation will require robust evidence demonstrating long‐term ecological safety, economic feasibility, regulatory compliance, and societal acceptance. As highlighted in Table 3, integration of multi‐omics datasets through systems‐level analytical frameworks provides a critical foundation for the rational design and optimization of future nanobiotic formulations.

Collectively, future progress in nanobiotic agriculture should be guided by six interconnected priorities: (i) mechanistic elucidation of plant–microbe–nanoparticle interactions; (ii) integration of multi‐omics and systems‐biology platforms; (iii) development of green, scalable, and economically viable nanoparticle synthesis strategies; (iv) long‐term field validation across diverse agroecosystems; (v) establishment of harmonized biosafety and regulatory frameworks; and (vi) application of artificial intelligence and predictive modeling for precision formulation design. Addressing these priorities through interdisciplinary collaboration among plant scientists, microbiologists, nanotechnologists, systems biologists, agronomists, economists, and regulatory stakeholders will be essential for transforming nanobiotic synergies into scientifically validated, environmentally responsible, and commercially viable technologies for sustainable agriculture.

## 11 Conclusion

The convergence of nanotechnology and rhizosphere microbiome science represents a promising frontier in sustainable agriculture, offering new opportunities to strengthen plant immunity, enhance stress resilience, and improve resource‐use efficiency. This review highlights how nanoparticles and beneficial rhizospheric microorganisms influence complementary biological processes, including immune priming, nutrient acquisition, antioxidant regulation, microbiome assembly, and adaptation to biotic and abiotic stresses. Emerging evidence suggests that integrating these components within a unified nanobiotic framework may provide multifunctional strategies capable of supporting crop health while reducing reliance on conventional chemical inputs. Despite encouraging progress, the field remains at an early stage of development. Significant knowledge gaps persist regarding the mechanistic basis of plant–microbe–nanoparticle interactions, the consistency of reported synergistic effects across diverse agroecosystems, and the long‐term ecological consequences of nanoparticle applications. Addressing these challenges will require a shift from predominantly descriptive studies toward mechanistic investigations supported by genomics, transcriptomics, proteomics, metabolomics, microbiome profiling, and systems‐level analyses. Equally important are rigorous field‐scale validations, comprehensive biosafety assessments, lifecycle evaluations, and harmonized regulatory frameworks to ensure environmental compatibility and societal acceptance. Future advances are likely to be driven by the integration of multi‐omics technologies, systems biology, artificial intelligence, and predictive modeling, enabling the rational design of crop‐specific and environmentally responsive nanobiotic formulations. By combining biological adaptability with advanced material functionality, nanobiotic technologies have the potential to contribute to climate‐resilient and resource‐efficient agricultural systems. Continued interdisciplinary collaboration among plant scientists, microbiologists, nanotechnologists, computational researchers, agronomists, and policymakers will be essential to translate this emerging concept into scientifically validated and practically deployable solutions for sustainable food production.

## Funding

The authors have nothing to report.

## Disclosure

The authors have nothing to report.

## Conflicts of Interest

The authors declare no conflicts of interest.

## Data Availability

Data sharing not applicable to this article as no datasets were generated or analyzed during the current study.

## References

[pei370183-bib-0001] Abd Alamer, I. S. , A. A. Tomah , T. Ahmed , B. Li , and J. Zhang . 2021. “Biosynthesis of Silver Chloride Nanoparticles by Rhizospheric Bacteria and Their Antibacterial Activity Against Phytopathogenic Bacterium *Ralstonia solanacearum* .” Molecules 27, no. 1: 224. 10.3390/molecules27010224. 35011455 PMC8746595

[pei370183-bib-0002] Abdelkhalek, A. , Y. Yassin , A. Abdel‐Megeed , K. A. Abd‐Elsalam , H. Moawad , and S. I. Behiry . 2022. “ *Rhizobium leguminosarum* bv. *viciae*‐Mediated Silver Nanoparticles for Controlling Bean Yellow Mosaic Virus (BYMV) Infection in Faba Bean Plants.” Plants 12, no. 1: 45. 10.3390/plants12010045. 36616172 PMC9823325

[pei370183-bib-0003] Ahmed, T. , M. Noman , J. L. Gardea‐Torresdey , J. C. White , and B. Li . 2023. “Dynamic Interplay Between Nano‐Enabled Agrochemicals and the Plant‐Associated Microbiome.” Trends in Plant Science 28, no. 11: 1310–1325. 10.1016/j.tplants.2023.06.001. 37453924

[pei370183-bib-0004] Al‐Harethi, A. A. , Q. Y. Abdullah , H. J. AlJobory , A. M. Anam , R. A. Arafa , and K. Y. Farroh . 2024. “Zinc Oxide and Copper Oxide Nanoparticles as a Potential Solution for Controlling *Phytophthora infestans* , the Late Blight Disease of Potatoes.” Discover Nano 19, no. 1: 105. 10.1186/s11671-024-04040-6. 38907852 PMC11193706

[pei370183-bib-0005] Amadou, I. , D. Houben , and M.‐P. Faucon . 2021. “Unravelling the Role of Rhizosphere Microbiome and Root Traits in Organic Phosphorus Mobilization for Sustainable Phosphorus Fertilization: A Review.” Agronomy 11, no. 11: 2267. 10.3390/agronomy11112267.

[pei370183-bib-0006] Argentel‐Martínez, L. , O. Peñuelas‐Rubio , A. Herrera‐Sepúlveda , et al. 2024. “Biotechnological Advances in Plant Growth‐Promoting Rhizobacteria for Sustainable Agriculture.” World Journal of Microbiology and Biotechnology 41, no. 1: 21. 10.1007/s11274-024-04231-4. 39738995

[pei370183-bib-0007] Asghar, W. , K. D. Craven , J. R. Swenson , R. Kataoka , A. Mahmood , and J. G. Farias . 2024. “Enhancing the Resilience of Agroecosystems Through Improved Rhizosphere Processes: A Strategic Review.” International Journal of Molecular Sciences 26, no. 1: 109. 10.3390/ijms26010109. 39795965 PMC11720004

[pei370183-bib-0008] Banerjee, S. , S. Ghosh , S. Jha , et al. 2025. “Enhancing Mycophytoremediation Potential of *Chrysopogon zizanioides* in Chromite‐Asbestos Mine Waste Soil Using Arbuscular Mycorrhizal Fungi: A Natural Bioaccelerator for Soil Ecosystem Rehabilitation.” Science of the Total Environment 989: 179884. 10.1016/j.scitotenv.2025.179884. 40513445

[pei370183-bib-0009] Banerjee, S. , S. Jha , S. Chakraborty , et al. 2025. “Mycorrhiza‐Assisted Phytoremediation of Spiked Chromium‐Contaminated Soil: Assessing AMF–Vetiver Symbiosis for Cr Accumulation and Soil Quality Enhancement.” Environmental Research 283: 122143. 10.1016/j.envres.2025.122143. 40517925

[pei370183-bib-0010] Begum, N. , L. Wang , H. Ahmad , et al. 2022. “Co‐Inoculation of Arbuscular Mycorrhizal Fungi and Plant Growth‐Promoting Rhizobacteria Improves Growth and Photosynthesis in Tobacco Under Drought Stress by Up‐Regulating Antioxidant and Mineral Nutrition Metabolism.” Microbial Ecology 83, no. 4: 971–988. 10.1007/s00248-021-01815-7. 34309697

[pei370183-bib-0011] Begum, N. , Y. Xiao , L. Wang , D. Li , A. Irshad , and T. Zhao . 2023. “ *Rhizophagus irregularis* Alleviates Drought Stress in Soybean Overexpressing the *GmSPL9d* Gene by Promoting Photosynthetic Apparatus and Regulating the Antioxidant System.” Microbiological Research 273: 127398. 10.1016/j.micres.2023.127398. 37167733

[pei370183-bib-0012] Behera, P. R. , K. K. Behera , G. Sethi , et al. 2024. “Enhancing Agricultural Sustainability Through Rhizomicrobiome: A Review.” Journal of Basic Microbiology 64, no. 11: e2400100. 10.1002/jobm.202400100. 38899609

[pei370183-bib-0013] Berruto, C. A. , and G. S. Demirer . 2024. “Engineering Agricultural Soil Microbiomes and Predicting Plant Phenotypes.” Trends in Microbiology 32, no. 9: 858–873. 10.1016/j.tim.2024.02.003. 38429182

[pei370183-bib-0014] Checcucci, A. , and M. Marchetti . 2020. “The Rhizosphere Talk Show: The Rhizobia on Stage.” Frontiers in Agronomy 2: 591494. 10.3389/fagro.2020.591494.

[pei370183-bib-0015] Chen, Y. , C. Sun , Y. Yan , D. Jiang , S. Huangfu , and L. Tian . 2025. “Impact of Arbuscular Mycorrhizal Fungi on Maize Rhizosphere Microbiome Stability Under Moderate Drought Conditions.” Microbiological Research 290: 127957. 10.1016/j.micres.2024.127957. 39486317

[pei370183-bib-0016] Chen, Y. , Z. Yao , Y. Sun , et al. 2022. “Current Studies of the Effects of Drought Stress on Root Exudates and Rhizosphere Microbiomes of Crop Plant Species.” International Journal of Molecular Sciences 23, no. 4: 2374. 10.3390/ijms23042374. 35216487 PMC8874553

[pei370183-bib-0017] Chen, Z. , R. Kama , Y. Cao , et al. 2025. “The Potential of Earthworms and Arbuscular Mycorrhizal Fungi to Enhance Phytoremediation in Heavy Metal‐Contaminated Soils: A Review.” Mycorrhiza 35, no. 3: 33. 10.1007/s00572-025-01207-6. 40272572

[pei370183-bib-0018] de Vries, F. T. , R. I. Griffiths , C. G. Knight , O. Nicolitch , and A. Williams . 2020. “Harnessing Rhizosphere Microbiomes for Drought‐Resilient Crop Production.” Science 368, no. 6488: 270–274. 10.1126/science.aaz5192. 32299947

[pei370183-bib-0019] Dlamini, S. P. , A. O. Akanmu , and O. O. Babalola . 2022. “Rhizospheric Microorganisms: The Gateway to Sustainable Plant Health.” Frontiers in Sustainable Food Systems 6: 925802. 10.3389/fsufs.2022.925802.

[pei370183-bib-0020] El‐Abeid, S. E. , M. A. Mosa , M. A. M. El‐Tabakh , A. M. Saleh , M. A. El‐Khateeb , and M. S. A. Haridy . 2024. “Antifungal Activity of Copper Oxide Nanoparticles Derived From *Zizyphus spina* Leaf Extract Against *Fusarium* Root Rot Disease in Tomato Plants.” Journal of Nanobiotechnology 22, no. 1: 28. 10.1186/s12951-023-02281-8. 38216982 PMC10785362

[pei370183-bib-0021] Elsharkawy, M. , A. Derbalah , A. Hamza , and A. El‐Shaer . 2020. “Zinc Oxide Nanostructures as a Control Strategy of Bacterial Speck of Tomato Caused by *Pseudomonas syringae* in Egypt.” Environmental Science and Pollution Research 27, no. 16: 19049–19057. 10.1007/s11356-018-3806-0. 30484042

[pei370183-bib-0022] Francioli, D. , G. Cid , S. Kanukollu , A. Ulrich , M. R. Hajirezaei , and S. Kolb . 2021. “Flooding Causes Dramatic Compositional Shifts and Depletion of Putative Beneficial Bacteria on the Spring Wheat Microbiota.” Frontiers in Microbiology 12: 773116. 10.3389/fmicb.2021.773116. 34803993 PMC8602104

[pei370183-bib-0023] Gatasheh, M. K. , A. A. Shah , M. Kaleem , S. Usman , and S. Shaffique . 2024. “Application of CuNPs and AMF Alleviates Arsenic Stress by Encompassing Reduced Arsenic Uptake Through Metabolomics and Ionomics Alterations in *Elymus sibiricus* .” BMC Plant Biology 24, no. 1: 667. 10.1186/s12870-024-05359-z. 38997682 PMC11245830

[pei370183-bib-0555] Ge, A. H. , and E. Wang . 2025. “Exploring the Plant Microbiome: A Pathway to Climate‐Smart Crops.” Cell 188, no. 6: 1469–1485. 10.1016/j.cell.2025.01.035. 40118032

[pei370183-bib-0024] Guan, X. , X. Gao , A. Avellan , et al. 2020. “CuO Nanoparticles Alter the Rhizospheric Bacterial Community and Local Nitrogen Cycling for Wheat Grown in a Calcareous Soil.” Environmental Science & Technology 54, no. 14: 8699–8709. 10.1021/acs.est.0c00036. 32579348

[pei370183-bib-0025] Han, Y. , X. Lou , W. Zhang , T. Xu , and M. Tang . 2022. “Arbuscular Mycorrhizal Fungi Enhanced Drought Resistance of *Populus cathayana* by Regulating the 14‐3‐3 Family Protein Genes.” Microbiology Spectrum 10, no. 3: e02456‐21. 10.1128/spectrum.02456-21. PMC9241863 35612316

[pei370183-bib-0222] Huang, D. , M. Ma , Q. Wang , et al. 2020. “Arbuscular Mycorrhizal Fungi Enhanced Drought Resistance in Apple by Regulating Genes in the MAPK Pathway.” Plant Physiology and Biochemistry: PPB 149: 245–255. 10.1016/j.plaphy.2020.02.020. 32087536

[pei370183-bib-0026] Islam, M. F. , S. Islam , M. A. S. Miah , et al. 2024. “Green Synthesis of Zinc Oxide Nanoparticles Using *Allium cepa* L. Waste Peel Extracts and Its Antioxidant and Antibacterial Activities.” Heliyon 10, no. 3: e25430. 10.1016/j.heliyon.2024.e25430. 38333859 PMC10850583

[pei370183-bib-0027] Jiao, L. , X. Cao , C. Wang , et al. 2023. “Crosstalk Between In Situ Root Exudates and Rhizobacteria to Promote Rice Growth by Selenium Nanomaterials.” Science of the Total Environment 878: 163175. 10.1016/j.scitotenv.2023.163175. 37003329

[pei370183-bib-0028] Jiao, L. , X. Cao , C. Wang , et al. 2025. “Deciphering the Dynamic Interplay Between Rhizobacteria and Root Exudates via Cerium Oxide Nanomaterials Modulation for Promoting Soybean Yield and Quality.” Journal of Agricultural and Food Chemistry 73, no. 6: 3413–3426. 10.1021/acs.jafc.4c11178. 39881521

[pei370183-bib-0029] Kumar, P. 2021. “Soil Applied Glycine Betaine With Arbuscular Mycorrhizal Fungi Reduces Chromium Uptake and Ameliorates Chromium Toxicity by Suppressing Oxidative Stress in Three Genetically Different *Sorghum bicolor* L. Cultivars.” BMC Plant Biology 21, no. 1: 336. 10.1186/s12870-021-03113-3. 34261429 PMC8281485

[pei370183-bib-0030] Kumari, A. , A. K. Gupta , S. Sharma , et al. 2024. “Nanoparticles as a Tool for Alleviating Plant Stress: Mechanisms, Implications, and Challenges.” Plants 13, no. 11: 1528. 10.3390/plants13111528. 38891334 PMC11174413

[pei370183-bib-0031] Liu, H. , Y. Zhang , L. Zhang , Y. Liu , Y. Chen , and Y. Shi . 2025. “Nano‐Selenium Strengthens Potato Resistance to Potato Scab Induced by *Streptomyces* spp., Increases Yield, and Elevates Tuber Quality by Influencing Rhizosphere Microbiomes.” Frontiers in Plant Science 16: 1523174. 10.3389/fpls.2025.1523174. 39963528 PMC11830815

[pei370183-bib-0032] Malgioglio, G. , G. F. Rizzo , S. Nigro , et al. 2022. “Plant–Microbe Interaction in Sustainable Agriculture: The Factors That May Influence the Efficacy of PGPM Application.” Sustainability 14, no. 4: 2253. 10.3390/su14042253.

[pei370183-bib-0033] Montoya‐Martínez, A. C. , F. I. Parra‐Cota , and S. de Los Santos‐Villalobos . 2022. “Beneficial Microorganisms in Sustainable Agriculture: Harnessing Microbes' Potential to Help Feed the World.” Plants 11, no. 3: 372. 10.3390/plants11030372. 35161353 PMC8839818

[pei370183-bib-0034] Muhammad, M. , A. Wahab , A. Waheed , et al. 2025. “Navigating Climate Change: Exploring the Dynamics Between Plant–Soil Microbiomes and Their Impact on Plant Growth and Productivity.” Global Change Biology 31, no. 2: e70057. 10.1111/gcb.70057. 39924996

[pei370183-bib-0035] Neme, K. , A. Nafady , S. Uddin , and Y. B. Tola . 2021. “Application of Nanotechnology in Agriculture, Postharvest Loss Reduction and Food Processing: Food Security Implication and Challenges.” Heliyon 7, no. 12: e08539. 10.1016/j.heliyon.2021.e08539. 34934845 PMC8661015

[pei370183-bib-0036] Niazi, F. , M. Ali , U. Haroon , et al. 2023. “Effect of Green Fe_2_O_3_ Nanoparticles in Controlling *Fusarium* Fruit Rot Disease of Loquat in Pakistan.” Brazilian Journal of Microbiology 54, no. 3: 1341–1350. 10.1007/s42770-023-01050-x. 37400611 PMC10484849

[pei370183-bib-0037] Nie, X. , Z. Zhao , X. Zhang , D. A. Bastías , Z. Nan , and C. Li . 2024. “Endophytes Alleviate Drought‐Derived Oxidative Damage in *Achnatherum inebrians* Through Increasing Antioxidants and Regulating Host Stress Responses.” Microbial Ecology 87, no. 1: 73. 10.1007/s00248-024-02391-2. 38758374 PMC11101377

[pei370183-bib-0038] Prakash, V. , S. Tripathi , P. Rai , et al. 2024. “Effect of Engineered Nanoparticles on Rhizospheric Microbes.” In Microbial Biotechnology for Sustainable Agriculture, Volume 2. Microorganisms for Sustainability, edited by N. K. Arora and B. Bouizgarne , vol. 34. Springer. 10.1007/978-981-97-2355-3_3.

[pei370183-bib-0039] Rahman, M. S. , M. N. I. Bhuiyan , M. Rahman , et al. 2025. “Advancements in Nanotechnology for Arsenic Remediation in Agricultural Systems: Challenges and Prospects.” Plant Nano Biology 13: 100169. 10.1016/j.plana.2025.100169.

[pei370183-bib-0040] Rajput, V. D. , A. Singh , T. Minkina , et al. 2021. “Nano‐Enabled Products: Challenges and Opportunities for Sustainable Agriculture.” Plants 10, no. 12: 2727. 10.3390/plants10122727. 34961197 PMC8707238

[pei370183-bib-0041] Saeed, Q. , X. Wang , F. U. Haider , et al. 2021. “Rhizosphere Bacteria in Plant Growth Promotion, Biocontrol, and Bioremediation of Contaminated Sites: A Comprehensive Review of Effects and Mechanisms.” International Journal of Molecular Sciences 22, no. 19: 10529. 10.3390/ijms221910529. 34638870 PMC8509026

[pei370183-bib-0042] Shah, A. A. , S. Usman , Z. Noreen , et al. 2024. “Fullerenol Nanoparticles and AMF Application for Optimization of *Brassica napus* L. Resilience to Lead Toxicity Through Physio‐Biochemical and Antioxidative Modulations.” Scientific Reports 14, no. 1: 30992. 10.1038/s41598-024-82086-3. 39730765 PMC11681114

[pei370183-bib-0043] Shah, T. , H. Khan , A. Ali , et al. 2024. “Silicon and Arbuscular Mycorrhizal Fungi Alleviate Chromium Toxicity in *Brassica rapa* by Regulating Cr Uptake, Antioxidant Defense Expression, the Glyoxalase System, and Secondary Metabolites.” Plant Physiology and Biochemistry 206: 108286. 10.1016/j.plaphy.2023.108286. 38169223

[pei370183-bib-0044] Shah, T. , Z. Khan , T. A. Alahmadi , et al. 2024. “Mycorrhizosphere Bacteria Inhibit Chromium Uptake and Phytotoxicity by Regulating Proline Metabolism, Antioxidant Defense System, and Aquaporin Gene Expression in Tomato.” Environmental Science and Pollution Research 31, no. 17: 24836–24850. 10.1007/s11356-024-32755-7. 38456983

[pei370183-bib-0045] Sun, R. Z. , Y. Y. Wang , X. Q. Liu , Z. L. Yang , and X. Deng . 2024. “Structure and Dynamics of Microbial Communities Associated With the Resurrection Plant *Boea hygrometrica* in Response to Drought Stress.” Planta 260, no. 1: 24. 10.1007/s00425-024-04459-2. 38858226

[pei370183-bib-0046] Thepbandit, W. , and D. Athinuwat . 2024. “Rhizosphere Microorganisms Supply Availability of Soil Nutrients and Induce Plant Defense.” Microorganisms 12, no. 3: 558. 10.3390/microorganisms12030558. 38543610 PMC10975764

[pei370183-bib-0047] Wahab, A. , M. Muhammad , A. Munir , et al. 2023. “Role of Arbuscular Mycorrhizal Fungi in Regulating Growth, Enhancing Productivity, and Potentially Influencing Ecosystems Under Abiotic and Biotic Stresses.” Plants 12, no. 17: 3102. 10.3390/plants12173102. 37687353 PMC10489935

[pei370183-bib-0048] Wang, Z. , J. Lian , J. Liang , et al. 2024. “Arbuscular Mycorrhizal Symbiosis Modulates Nitrogen Uptake and Assimilation to Enhance Drought Tolerance of *Populus cathayana* .” Plant Physiology and Biochemistry 210: 108648. 10.1016/j.plaphy.2024.108648. 38653094

[pei370183-bib-0049] Wang, Z. , S. Zhang , J. Liang , et al. 2024. “ *Rhizophagus irregularis* Regulates *RiCPSI* and *RiCARI* Expression to Influence Plant Drought Tolerance.” Plant Physiology 197, no. 1: kiae645. 10.1093/plphys/kiae645. 39657034

[pei370183-bib-0050] Wang, Z. Y. , Y. J. Zhong , Y. F. Wang , et al. 2025. “Ecological Functions of Plant‐Beneficial Microbiomes and Their Application Prospects in Sustainable Agriculture.” Ying Yong Sheng Tai Xue Bao (Journal of Applied Ecology) 36, no. 5: 1553–1566. 10.13287/j.1001-9332.202504.036. 40456658

[pei370183-bib-0051] Wei, D. , X. Zhang , Y. Guo , et al. 2025. “CuO Nanoparticles Facilitate Soybean Suppression of Fusarium Root Rot by Regulating Antioxidant Enzymes, Isoflavone Genes, and Rhizosphere Microbiome.” Plant Physiology and Biochemistry 222: 109788. 10.1016/j.plaphy.2025.109788. 40096759

[pei370183-bib-0052] Zhang, H. , T. Zheng , Y. Wang , T. Li , and Q. Chi . 2024. “Multifaceted Impacts of Nanoparticles on Plant Nutrient Absorption and Soil Microbial Communities.” Frontiers in Plant Science 15: 1497006. 10.3389/fpls.2024.1497006. 39606675 PMC11600800

